# 2506 Post-discharge opioid prescriptions and their association with healthcare utilization in the Vanderbilt Inpatient Cohort Study

**DOI:** 10.1017/cts.2018.298

**Published:** 2018-11-21

**Authors:** Justin Scott Liberman, Lauren R. Samuels, Kathryn M. Goggins, Sunil Kripalani, Christianne Roumie

**Affiliations:** Vanderbilt University Medical Center

## Abstract

OBJECTIVES/SPECIFIC AIMS: Opioid prescribing is common and increasing in certain areas of the country with known risk of misuse and dependence. Our study examined the association of opioid prescription at discharge after hospitalization for acute coronary syndrome (ACS) or acute decompensated heart failure (ADHF) with emergency department (ED) care or all-cause readmission, intended healthcare utilization (follow-up with physician within 30 d of discharge and cardiac rehab participation), and all-cause mortality. METHODS/STUDY POPULATION: The Vanderbilt Inpatient Cohort Study is a prospective cohort of hospitalized patients age >18 enrolled with either ACS or ADHF between 2011 and 2015 (index hospitalization). We then excluded those who died during the index hospitalization, patients with hospitalization <24 hours, patients discharged to hospice care, or those who underwent coronary artery bypass surgery because of the high probability of receiving opioids. In addition, we limited the analyses to patients whom we had complete covariate data. The primary predictor variable was an opioid prescription at the time of hospital discharge. We collected healthcare utilization behavior for 90 days after discharge, and mortality data until March 8, 2017. Time-to-event analysis using Cox proportional hazard models was performed for both unintended healthcare utilization behavior and mortality outcomes. Logistic regression was performed for intended healthcare utilization (adherence to follow-up appointments and cardiac rehabilitation). All models were adjusted for demographic data, opioid use prior to index hospitalization, severity of illness, and healthcare utilization prior to the index hospitalization. RESULTS/ANTICIPATED RESULTS: There were 501 patients discharged with an opioid prescription and 1994 with no opioid prescription at discharge. Among patients with opioids at discharge 235 (47%) experienced unplanned healthcare events (71 ED visits and 164 readmissions) and among nonopioids patients 775 (39%) experienced unplanned healthcare events (254 ED visits and 521 readmissions) (aHR: 1.06, 95% CI: 0.87, 1.28). Patient mortality in the opioid group was 131 Versus 432 in the nonopioid group (aHR: 1.08, 95% CI 0.84, 1.39). Patients in the opioid at discharge group were less likely to attend follow up visits or participate in cardiac rehab (OR: 0.69, 95% CI 0.52, 0.91, *p*=0.009) compared with those not discharged on opioid medications. Sensitivity analysis of patients who were prescribed prehospital opioids (including prehospital opioids in the exposure group with postdischarge opioids) did not reveal a statistically significant increase in mortality (aHR: 1.09, 95% CI 0.91, 1.31) or unintended healthcare utilization (aHR: 1.12, 95% CI 0.89, 1.41) among opioid users. DISCUSSION/SIGNIFICANCE OF IMPACT: Morbidity and mortality related to opioid use is a public health concern. Our study demonstrates a statistically significant reduction in physician follow-up and participation in cardiac rehab among opioid users, both of which are known to decrease patient mortality. We did not find a statistically significant increase in unplanned healthcare utilization or mortality. Sensitivity analysis combining prehospital and posthospital opioid prescriptions did not reveal a statistically significant association between opioid use, hospital readmissions, or mortality. The hospital provides unique patient interactions where providers can make significant medical changes based on their patient’s clinical status. Continuing to understand the association between opioid use, healthcare utilization, morbidity, and mortality in recently hospitalized cardiac patients will provide data to support reduction in total opioid dose to improve clinical outcomes.

